# Supplementary material for: Atrial Fibrillation: A New Indicator for Advanced Colorectal Neoplasia in Screening Colonoscopy
Source: J Clin Med. 2019 Jul 23;8(7):1083. doi: 10.3390/jcm8071083 (PMC6678897; doi:10.3390/jcm8071083)
Supplement: Supplementary file 1 [file jcm-08-01083-s001.pdf]

Supplementary Table 1. Additional Findings on Colonoscopy.

	All Participants	Non-AF Group			AF Group	Statistical Comparison AF vs. Non-AF		
		Unmatched	1:1 Matched	3:1 Matched		Unmatched	1:1Matched	3:1Matched
**Number of Participants**	**1949**	**1903**	**46**	**138**	**46**	**-**	**-**	**-**
ANY COLONIC LESION*								
Number of Colonic Lesion per Participant								
0	1366 (70.1%)	1349 (70.9%)	34 (73.9%)	93 (67.4%)	17 (37.0%)	**<0.001**	**0.005**	**<0.001**
1	358 (18.4%)	342 (18.0%)	7 (15.2%)	27 (19.6%)	16 (34.8%)			
≥ 2	224 (11.5%)	211 (11.1%)	5 (10.9%)	19 (13.8%)	13 (28.2%)			
Size of Largest Colonic Lesion per Participant								
< 5 mm	462 (23.7%)	450 (23.6%)	7 (15.2%)	36 (26.0%)	13 (28%)	**<0.001**	**0.021**	**0.017**
≥ 5 - <10 mm	107 (5.5%)	98 (5.1%)	8 (17.4%)	14 (10.1%)	10 (21.7%)			
≥ 10 mm	60 (3.1%)	56 (2.9%)	0	2 (1.4%)	4 (8.7%)			
ADENOMAS								
Number of Tubular Adenomas per Participant								
0	1444 (74.1%)	1415 (74.4%)	34 (73.9%)	98 (71.0%)	29 (63.0%)	**<0.001**	0.231	0.058
1	316 (16.2%)	309 (16.2%)	7 (15.2%)	26 (18.8%)	7 (15.2%)			
≥ 2	188 (9.6%)	178 (9.4%)	5 (10.9%)	14 (10.1%)	10 (21.7%)			
Number of Advanced Adenomas per Participant								
0	1867 (95.8%)	1823 (95.8%)	46	134 (97.1%)	44 (95.7%)	0.067	0.360	0.215

1	70 (3.6%)	69 (3.6%)	0	4 (2.9%)	1 (2.2%)			
2	6 (0.3%)	6 (0.3%)	0	0	0			
3	5 (0.3%)	4 (0.2%)	0	0	1 (2.2%)			
DIVERTICULOSIS								
Severity								
None	1152 (59.1%)	1131 (59.4%)	25 (54.3%)	66 (75.9%)	21 (45.7%)	**0.010**	0.507	0.275
Mild	421 (21.6%)	409 (21.5%)	10 (21.7%)	33 (23.9%)	12 (26.1%)			
Intermediate	142 (7.3%)	141 (7.4%)	3 (6.5%)	14 (10.1%)	1 (2.2%)			
Severe	234 (12.0%)	222 (11.7%)	8 (17.4%)	25 (18.1%)	12 (26.1%)			
Extent of Diverticulosis								
Sigmoid Only	475 (25.2%)	461 (59.9%)	13 (61.9%)	43 (59.7%)	14 (56.0%)	**0.049**	0.664	0.627
> Sigmoid	99 (5.2%)	93 (12.1%)	3 (14.3%)	10 (13.9%)	6 (24.0%)			
Whole Colon Affected	221 (11.7%)	216 (28.0%)	5 (23.8%)	19 (26.4%)	5 (20%)			

Numbers are given as number (percentage). The level of significance was set at 0.05 and significant differences are marked in bold. *including hyperplastic polyps, adenoma, advanced neoplasia.
